# Ship Motion Attitude Prediction Model Based on FMD-IBKA-BTGN

**DOI:** 10.3390/s25216602

**Published:** 2025-10-27

**Authors:** Chunyuan Shi, Yanguan Su, Biao Zhang

**Affiliations:** 1 School of Mechanical and Power Engineering, Harbin University of Science and Technology, Harbin 150080, China; 2 School of Automation, Harbin University of Science and Technology, Harbin 150080, China; su_yan_guan@163.com (Y.S.); zhangbiao@hrbust.edu.cn (B.Z.)

**Keywords:** ship motion attitude prediction, FMD, IBKA, Bi-TCN, Bi-GRU

## Abstract

Accurate prediction of ship motion attitude remains a significant challenge due to the inherent non-stationarity and strong stochasticity of marine environmental conditions. To address this issue, this study proposes FMD-IBKA-BTGN, a hybrid model combining Feature Mode Decomposition (FMD), Improved Black-winged Kite Algorithm (IBKA), and a Bidirectional Temporal Convolutional Network with Gated Recurrent Unit (BTGN). First, FMD decomposes motion signals into intrinsic modes. Subsequently, IBKA—enhanced with chaotic mapping and Lévy flights—optimizes BTGN hyperparameters for global search efficiency. Finally, predictions from all components are ensembled for final output. Experiments on a 240 m vessel in Sea State 4 show our model outperforms six models, reducing MAPE by 20.38%, RMSE by 7.4%, MAE by 4.2%, and MSE by 0.97% versus LSTM. The model enhances both prediction accuracy and generalization.

## 1. Introduction

The safe navigation of ships is essential for the maritime industry and the safety of crew and cargo. It remains a key research focus in naval architecture and ocean engineering. Under wind and wave actions, ships undergo six degrees of freedom motions: roll, pitch, yaw, sway, surge, and heave. Among these, roll, pitch, and heave have the most critical influence on ship behavior. Excessive roll can compromise stability, potentially leading to capsizing, and may damage onboard equipment. Severe pitch and heave motions can induce propeller emergence racing and green water on deck—both degrade propulsion efficiency, reduce speed, and complicate maneuvering. Therefore, accurate prediction of future ship motions enables proactive compensation strategies, enhancing operational safety and preventing hazardous events such as anchoring failures or capsizing [1].

Ship motion attitude prediction involves establishing time-series models based on historical attitude data to forecast future trends. Early studies relied on statistical analysis and dynamic modeling, such as the Kalman filter [2], but these methods struggled with complex mathematical formulations and computational complexity, limiting their practical application. Subsequently, linear models like autoregressive [3], autoregressive moving average [4], and autoregressive integrated moving average were adopted [5]. While these models demonstrated adaptability, they faced limitations in handling nonlinear data.

With the rise in artificial intelligence, machine learning [6] and deep learning [7] techniques have been increasingly applied to ship motion prediction. Models such as support vector machines and backpropagation neural networks offered rapid responses and strong fitting capabilities but struggled to adapt to complex data patterns. Recurrent neural networks, LSTM, GRU, and TCN [8,9,10] emerged as alternatives due to their ability to process nonlinear sequences. However, single-network models often lack robustness in noisy or dynamic environments. Recent research has shifted toward hybrid models to address these challenges.

Hybrid models primarily include three aspects: improving single-model architectures, data preprocessing, and optimizing model parameters using swarm intelligence algorithms [11,12]. Improving single models mainly involves hybridizing multiple models. For instance, Su proposed a real-time ship acceleration prediction algorithm combining LSTM and GRU networks [13]. Additionally, a hybrid LSTM-CNN model was constructed for roll motion forecasting, demonstrating superior performance compared to single-model frameworks [14]. Zhao et al. proposed a new method for enhancing Fourier transform and multilayer perceptron network based on DeepONet. This method effectively captures and learns the motion patterns of ships in both time and frequency domains [15]. Beyond model architecture improvements, selecting appropriate decomposition methods to split data into components can reduce prediction complexity and enhance accuracy. Techniques such as wavelet decomposition, empirical mode decomposition, ensemble EMD, and variational mode decomposition have been integrated into ship motion prediction models [16,17,18]. While these methods adaptively decompose input signals into intrinsic mode functions, they suffer from mode mixing and limited adaptability. FMD decomposes the raw ship-motion attitude data into a set of intrinsic mode functions while simultaneously capturing both the impulsive and periodic nature of the data. Leveraging the merits of adaptive filter updating and period estimation, it can accurately extract the underlying information even under complex conditions. Therefore, Miao introduced Feature Mode Decomposition, which simultaneously considers impulse and periodic characteristics of data, offering a novel preprocessing strategy [19].

In addition to data preprocessing, optimizing model parameters via swarm intelligence algorithms can further improve prediction performance. Studies show that parameter configurations significantly impact model outcomes. For example, Zhang improved the Whale Optimization Algorithm to optimize TCN hyperparameters for ship motion prediction [20]. The Black-winged Kite Algorithm [21,22], a novel swarm optimization method inspired by avian foraging and migration behaviors, has been widely applied in engineering optimization. However, traditional BKA struggles with slow convergence and premature stagnation. To resolve this, Zhang proposed an improved heuristic algorithm using logistic chaotic mapping for population initialization, enhancing search efficiency [23]. Experimental results demonstrate that IBKA outperforms conventional BKA in ship motion prediction tasks.

Existing deep learning models often emphasize forward features but may struggle to track dynamic changes in ship motion, limiting their predictive capabilities. Li developed a new ship motion prediction model using Bi-LSTM and improved WOA, validating the advantages of bidirectional structures and hybrid models [24]. Gao employed bidirectional residual gate recurrent units and bidirectional residual long short-term memory to form residual blocks for real-time ship motion forecasting [25]. To address the nonlinearity and stochasticity of ship motion data, this study proposes a novel prediction method combining bidirectional feature extraction, FMD, and IBKA optimization:

First, FMD preprocesses ship motion data by decomposing it into distinct components. A BTGN model is then applied to each component for individual data modeling. Finally, predictions from all components are aggregated to produce the overall result. Concurrently, IBKA optimizes the parameters of the BTGN to achieve optimal model performance.

The main contributions of this work are as follows:

1. The BTGN model integrates bidirectional TCN and GRU networks, effectively capturing both forward and backward temporal dynamics to enhance prediction accuracy.
2. FMD simultaneously accounts for impulsive and periodic characteristics in the data, enabling more thorough decomposition and thereby improving the learning capability of BTGN.
3. IBKA incorporates dual chaotic mapping and Lévy flight strategies to promote global convergence, effectively tuning BTGN parameters and enhancing the performance of the hybrid model.

The remainder of this paper is organized as follows: Section 2 introduces FMD, BTGN architecture, and IBKA optimization mechanisms. Section 3 proposes the FMD-IBKA-BTGN prediction framework and experimental design. Section 4 presents experimental results and comparative analysis. Section 5 summarizes findings and discusses future research directions.

## 2. Methods

In this section, the basic theories of the model are detailed, including FMD, BTGN model implementation for bidirectional feature extraction, and the improved process of the Black-winged Kite Algorithm.

### 2.1. Data Decomposition

FMD is a non-recursive decomposition method that simultaneously considers the impulse and periodic characteristics of ship motion data. This method leverages the advantages of filter updates and period estimation, enabling accurate decomposition of data information even in complex scenarios. Based on deconvolution theory, an adaptive FIR filter is designed by initializing an FIR filter bank with Hann windows and iteratively updating filter coefficients, such that the filtered signal infinitely approaches the deconvolution target function. Additionally, FMD has been proven to have strong robustness against noise interference. The main stages of using FMD to decompose ship motion data include:

(1) Initialize the FIR filter bank with Hann windows

**Figure 1:**
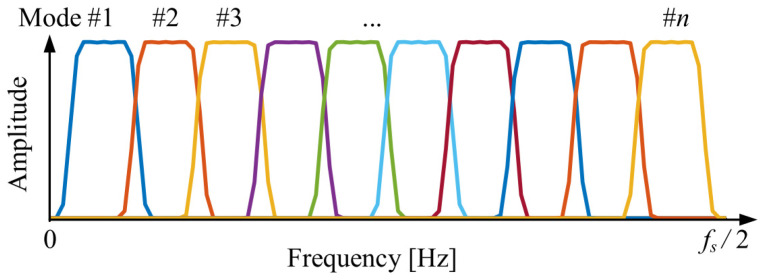
FIR filter bank initialized through Hann window.

First, the frequency band of the original signal is uniformly divided into *K* segments. Then, the upper cutoff frequency $f_{u}$ and lower cutoff frequency $f_{l}$ of these segments are specified as:

(1) $$\left\{\begin{array}{c} f_{l}=k\cdot f_{s}/2K\\ f_{u}=(k+1)\cdot f_{s}/2K \end{array}\right.\;k=0,1,2,\ldots ,K-1$$

where $f_{s}$ is the sampling frequency of the original signal. As shown in Figure 1, displayed in the Hann window, A set of uniformly distributed FIR filters that cover the entire frequency band can form an FIR filter bank.

(2) Updating the filter and period

Select *CK* as the target function to update the filter. The original signal of length *N* is labeled as $x\left(n\right)$, with the following constraints:

(2) $$\underset{\left\{f_{k}(l)\right\}}{\arg\;\max}\left\{CK_{M}(u_{k})\;=\;\sum_{n=1}^{N}{\left(\prod_{m=0}^{M}u_{k}\left(n-mT_{s}\right)\right)}^{2}\;/\;{\left(\sum_{n=1}^{N}u_{k}{\left(n\right)}^{2}\right)}^{M+1}\right\}$$

(3) $$s.t.u_{k}\left(n\right)=\sum_{l=1}^{L}f_{k}(l)\;x(n-l+1)$$

where $u_{k}\left(n\right)$ represents the *k*-th decomposition mode. $f_{k}$ denotes the *k*-th FIR filter with length *L*, where $T_{s}$ is the input period measured in terms of the number of samples. *M* is the shift order. The filter coefficients are continuously updated through the solution of the constrained problem to approach the target setting. Subsequently, the period is estimated using the autocorrelation spectrum, where the signal’s autocorrelation spectrum produces local maxima at periodic positions. The FIR filter is updated, and the estimated period is refined iteratively, gradually achieving higher accuracy.

(3) Mode Selection

To eliminate unnecessary mode mixing and redundancy, a K×K matrix *CC* is constructed using two modes. Subsequently, the two modes with the maximum *CC* values *CC*max are locked, and their CK values are calculated using the estimated period. Then, the mode with the smaller CK is discarded from the two modes. The two modes $u_{p}$ and $u_{q}$ are defined as:

(4) $$CC_{pq}=\frac{\sum_{n=1}^{N}\left(u_{p}(n)-{\bar{u}}_{p})(u_{q}(n)-{\bar{u}}_{q}\right)}{\sqrt{\sum_{n=1}^{N}{\left(u_{p}(n)-{\bar{u}}_{p}\right)}^{2}}\sqrt{\sum_{n=1}^{N}{\left(u_{q}(n)-{\bar{u}}_{q}\right)}^{2}}}$$

where ${\bar{u}}_{p}$ and ${\bar{u}}_{q}$ are, respectively, the mean of mode $u_{p}$ and $u_{q}$.

(4) Signal Reconstruction

FMD decomposes the original signal into several intrinsic mode functions. Calculate the permutation entropy-kurtosis ratio for each IMF after decomposition. Remove the IMF with the highest PEK value and complete signal reconstruction.

### 2.2. Hybrid Bidirectional Temporal Convolutional Network and Bidirectional Gated Recurrent Unit Model

#### 2.2.1. Bi-TCN

Unlike traditional RNNs and CNNs, TCN uses one-dimensional convolution operations to build a deep temporal feature extractor. TCN retains the powerful feature extraction capabilities of traditional neural networks while also better capturing long-term dependencies in sequences. TCN is primarily composed of three components: residual connections, causal convolutions, and dilated convolutions. In sequence modeling, TCN is based on two principles: maintaining temporal causality and ensuring consistency between input and output dimensions. To implement these principles, a one-dimensional fully convolutional network (FCN) is used as the base architecture, combined with causal convolutions to ensure unidirectional information flow. In the FCN, the dimensions of the hidden layers are aligned with the input layer, and zero padding is used to maintain the same length across adjacent layers. If the input contains n time steps, the output will also have n time steps. Causal convolution is a unidirectional structure where the current time step depends only on the current and previous time steps, ensuring that future information does not leak into the current state. To meet the need for capturing long-term dependencies and to avoid gradient vanishing caused by increasing convolution depth, dilated convolutions and residual connections are employed. Dilated convolutions are used to achieve an exponentially large receptive field, enabling the processing of long-term historical information. Dilated convolution is defined as:

(5) $$F(s)=(X{*}_{d}f)(s)=\sum_{i=0}^{k-1}f(i)\cdot X_{s-d\cdot i}$$

where ∗ represents the convolution operation, d represents the dilation factor, f denotes the filter size, and s−d⋅i indicates the past direction.

**Figure 2:**
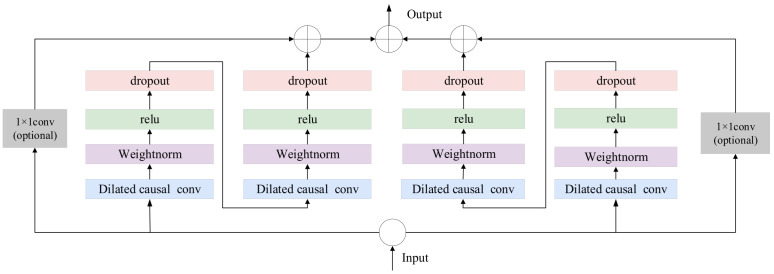
Bi-TCN Structure Diagram.

With the increase in network depth, issues such as gradient vanishing and degradation of model performance may arise, leading to the development of residual connection modules. In TCN, the residual connection module replaces the simple linking between layers. It contains a causal convolution, an activation function, and dropout. Additionally, to maintain consistent input and output widths of the module, an extra convolution is added to ensure the same shape. Residual connections between layers enable information propagation across different layers, avoiding gradient vanishing and exploding caused by increasing receptive fields. Bi-TCN, on the other hand, effectively captures the contextual information and long-term dependencies in ship motion attitude data by combining forward and backward temporal convolutional networks. Figure 2 illustrates the structure of Bi-TCN, where the forward TCN processes historical information, the backward TCN captures backward information features, and the final output is the merged output of both.

#### 2.2.2. Bi-GRU

**Figure 3:**
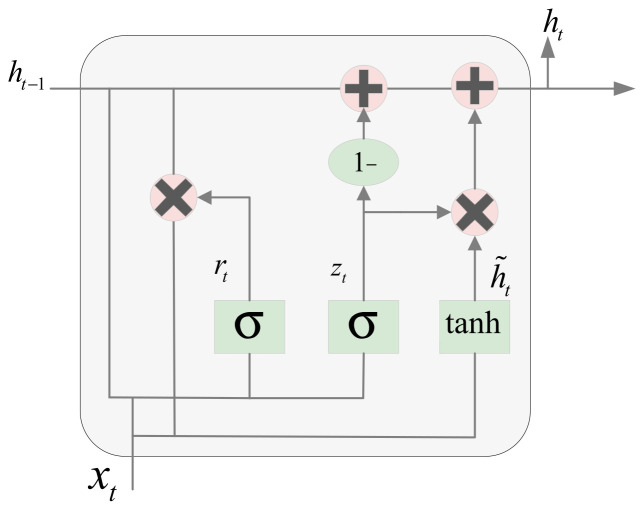
GRU Structure Diagram.

The Gated Recurrent Unit (GRU) is an improved version of the RNN, which combines the forget gate and input gate into a unified update gate unit, reducing model complexity and parameter count. GRU incorporates an update gate and a reset gate, and the calculations of the update gate and reset gate are shown in the formula:

(6) $$z_{t}=\sigma \left(W_{z}\cdot \left[h_{t-1},x_{t}\right]+b_{z}\right)$$

(7) $$r_{t}=\sigma \left(W_{r}\cdot \left[h_{t-1},x_{t}\right]+b_{r}\right)$$

where $W_{z}$ and $b_{z}$ are the parameter matrix and bias vector of the update gate, $W_{r}$ and $b_{r}$ are the parameter matrix and bias vector of the reset gate, $h_{t-1}$ is the hidden state from the previous time step, $x_{t}$ is the current input, and σ is the Sigmoid activation function. The update gate controls the amount of historical information retained from the previous state in the current time step calculation, while the reset gate determines the contribution degree of the candidate hidden state at the current time step to the final hidden state update. The formulas for the candidate hidden state and the final hidden state are as follows:

(8) $${\tilde{h}}_{t}=\tanh(W\cdot \left[r_{t}\cdot h_{t-1},x_{t}\right]+b)$$

(9) $$h_{t}=z_{t}\cdot h_{t-1}+\left(1-z_{t}\right)\cdot {\tilde{h}}_{t}$$

where $r_{t}\cdot h_{t-1}$ represents the combination of the previous time step’s hidden state $h_{t-1}$ with the reset gate $r_{t}$ to control its influence degree, and *W* is the matrix for candidate hidden state computation. Through this mechanism, GRU achieves dynamic filtering and updating of information while reducing model complexity, as illustrated in Figure 3.

**Figure 4:**
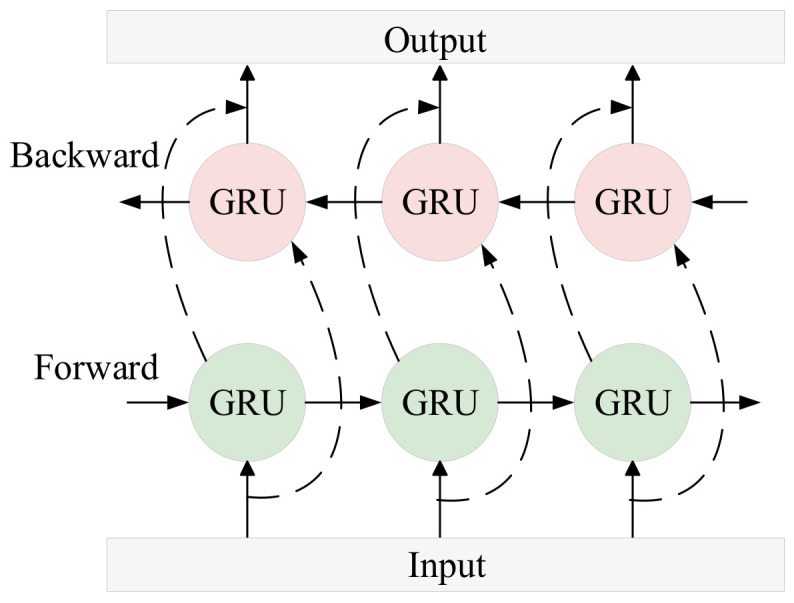
Bi-GRU Structure Diagram.

Bi-GRU consists of two independent GRU layers, one processing the sequence in the forward direction and the other in the backward direction. The model structure of Bi-GRU is shown in Figure 4. The calculation formulas for the forward and backward computations of the Bi-GRU model are as follows:

(10) $$h_{t}^{\left(f\right)}=GRU(x_{t},h_{t-1}^{\left(f\right)})$$

(11) $$h_{t}^{\left(b\right)}=GRU(x_{t},h_{t+1}^{\left(b\right)})$$

Concatenate the hidden states from both directions to obtain the final hidden state.

#### 2.2.3. BTGN Model

**Figure 5:**
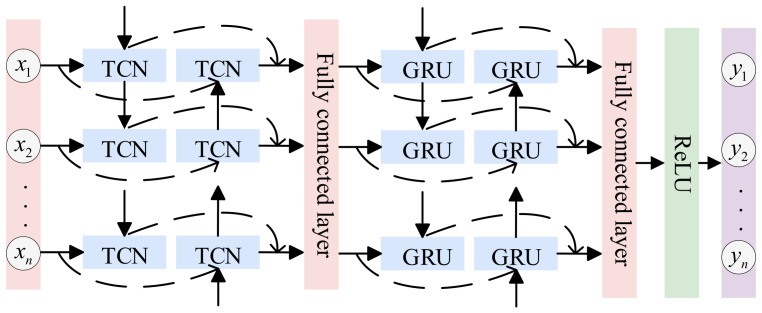
BTGN Structure Diagram.

The final hybrid model is shown in Figure 5. The Bi-TCN model employs forward and backward convolutional operations to extract contextual information from sequential data, forming a feature sequence. Subsequently, this sequence is fed into the Bi-GRU model. During the forward and backward updates of hidden states in the Bi-GRU model, GRUs capture long-range dependencies in the sequence and output a feature sequence integrating global information.

### 2.3. Improved Black Kite Algorithm

The Black-winged Kite Optimization Algorithm is a population-based intelligent algorithm inspired by the foraging behavior of birds. The algorithm is modeled based on the attacking and migrating behaviors of the black-winged kite. The attacking behavior can be regarded as the process of searching for the optimal solution within a specific range, where the prey corresponds to the global optimal solution in the algorithm. The migrating behavior is analogous to the algorithm’s exploration over a broader space, helping to avoid falling into local optima and enabling the search for new potential solution regions. The algorithmic process is divided into three stages: population initialization, the attacking phase, and the migration phase. However, the algorithm has a drawback in that it tends to fall into local optima in the later stages of iteration, leading to premature convergence and preventing the algorithm from achieving more precise solutions. To address the issue of low randomness in the initial population, this paper proposes a Logistic-Tent double chaotic mapping initialization method, which increases the probability of the initial population being closer to the optimal solution, thereby fundamentally improving the slow convergence speed. To tackle the problem of the standard BKA being prone to local optima, this study introduces a Levy flight strategy to enhance the algorithm’s global search capability, allowing the algorithm to escape from local optima. The basic principles are as follows:

(1) Population Initialization:

Create a matrix *BK* to represent the random positions of each black kite and uniformly distribute them using a formula.

(12) $$X_{i}=BK_{lb}+rand(BK_{ub}-BK_{lb})$$

where *i* is an integer between 1 and pop. Here, $BK_{ub}$ and $BK_{lb}$ represent the lower and upper bounds of the *j*-th dimension for the *i*-th black kite individual, respectively, and rand is a randomly selected value within the interval [0, 1].

Then, define a sequence $Z_{n}$ as follows:

(13) $$z_{n}=\left\{\begin{array}{ccc} \left[rz_{n-1}(1-z_{n-1})+\frac{(4-r)z_{n-1}}{2}\right]\mathrm{mod}1 & & z_{n-1}<0.5\\ \left[rz_{n-1}(1-z_{n-1})+\frac{\left(4-r)(1-z_{n-1}\right)}{2}\right]\mathrm{mod}1 & & z_{n-1}\geq 0.5 \end{array}\right.$$

where $Z_{n}\in \left(0,1\right)$ represents the mapping variable; $r\in \left(0,1\right)$ denotes the control parameter; mod1 indicates the modulo operation. Through the Logistic-Tent dual chaotic mapping, the population distribution becomes more uniform, and Formula (12) is updated as:

(14) $$X_{i}=BK_{lb}+z_{n}\times (BK_{ub}-BK_{lb})$$

In the initial population of BKA, the individual with the best fitness value is selected as the leader $X_{L}$, $X_{L}$ is considered the optimal position.

(15) $$f_{best}=\min(f(X_{i}))$$

(16) $$X_{L}=X\left(find\left(f_{best}==f\left(X_{i}\right)\right)\right)$$

(2) Attack Behavior

Simulating the predation behavior of black kites, where individuals observe and adjust flight paths to capture prey. The mathematical model is defined as:

(17) $$y_{t+1}^{i,j}=\left\{\begin{array}{ccc} y_{t}^{i,j}+n(1+\sin(r))\times y_{t}^{i,j} & & \;p<r\\ y_{t}^{i,j}+n\times (2r-1)\times y_{t}^{i,j} & & else \end{array}\right.,\;n=0.05\times e^{-2\times {\left(\frac{t}{T}\right)}^{2}}$$

where $y_{t}^{i.j}$ and $y_{t+1}^{i.j}$ represent the position of the *i*-th black kite individual in the *j*-th dimension during the *t*-th and (*t* + 1)-th iteration steps, respectively. *r* is a random number between 0 and 1, and *p* is a constant value of 0.9. *T* denotes the total number of iterations, while *t* represents the number of completed iterations so far.

(3) Migration Behavior

According to the characteristics of bird migration, an excellent leader dynamically selected to guide the population during migration. The leadership migration rules are as follows: If the fitness value of the current population is less than that of a randomly selected population, the leader will abandon its leadership role and join the migrating population. Conversely, if the fitness value of the current population is greater than that of a randomly selected population, the leader will continue to guide the population until it reaches its destination. Therefore, a Levy-based tangent flight strategy is proposed for situations where the fitness value of the current population is lower than that of the random population. This strategy helps reduce dependence on individuals that have lost their leadership roles, thereby enhancing the algorithm’s search capability and expanding its search scope.

The mathematical model for the migration behavior of the Black Kite is as follows:

(18) $$y_{t+1}^{i.j}=\left\{\begin{array}{cc} y_{t}^{i.j}\times L_{F}\times \tan(\theta )\times C(0,1)\times (y_{t}^{i.j}-L_{t}^{j})\;F_{i}<F_{ri} & \\ y_{t}^{i.j}+C(0,1)\times (L_{t}^{j}-m\times y_{t}^{i.j})\;\;else & m=2\times \sin(r+\pi /2) \end{array}\right.$$

where $L_{t}^{j}$ represents the leading scorer in the *j*-th dimension during the *t*-th iteration so far. $F_{i}$ denotes the current position of any black kite individual in the *j*-th dimension during the *t*-th iteration. $F_{ri}$ indicates the fitness value of a randomly selected position in the *j*-th dimension obtained from any black kite individual during the *t*-th iteration. $C\left(0,1\right)$ represents the Cauchy mutation. $L_{F}$ represents the Levy flight function, and $\tan\left(\theta \right)$ denotes the movement direction, enabling precise adjustments in local search. When the value of θ gradually approaches 0, the tangent slope decreases, corresponding to a smaller change in step size, resulting in solutions closer to the current solution—ideal for local search. Conversely, when θ approaches π/2, the tangent slope increases, leading to larger step size adjustments and solutions farther from the current state—suitable for global search. The IBKA pseudocode is shown in Algorithm 1.
**Algorithm 1:** Improved Black—Winged Kite AlgorithmInput: The population size pop, maximum number of iterations T, and variable dimension dim Output: The best quasi-optimal solution obtained by IBKA for a given optimization problem 1. Initialization phase 2. Initialize the population size using logistic-Tent chaotic mapping and calculate the target fitness value of the population 3. while (*t* < T)do 4. /*Attacking behavior*/ 5. The location update parameter is updated as shown in Formula (15) 6. /*Migration behavior*/ 7. The location update parameter is updated as shown in Formula (16) 8. /*Select the best individual*/ 9. Check if it exceeds the search space and calculate fitness 10. Update individual optimal position if there is a better solution 11. *t* = *t* + 1 12. End while 13. Return best position and fitness value

**Figure 6:**
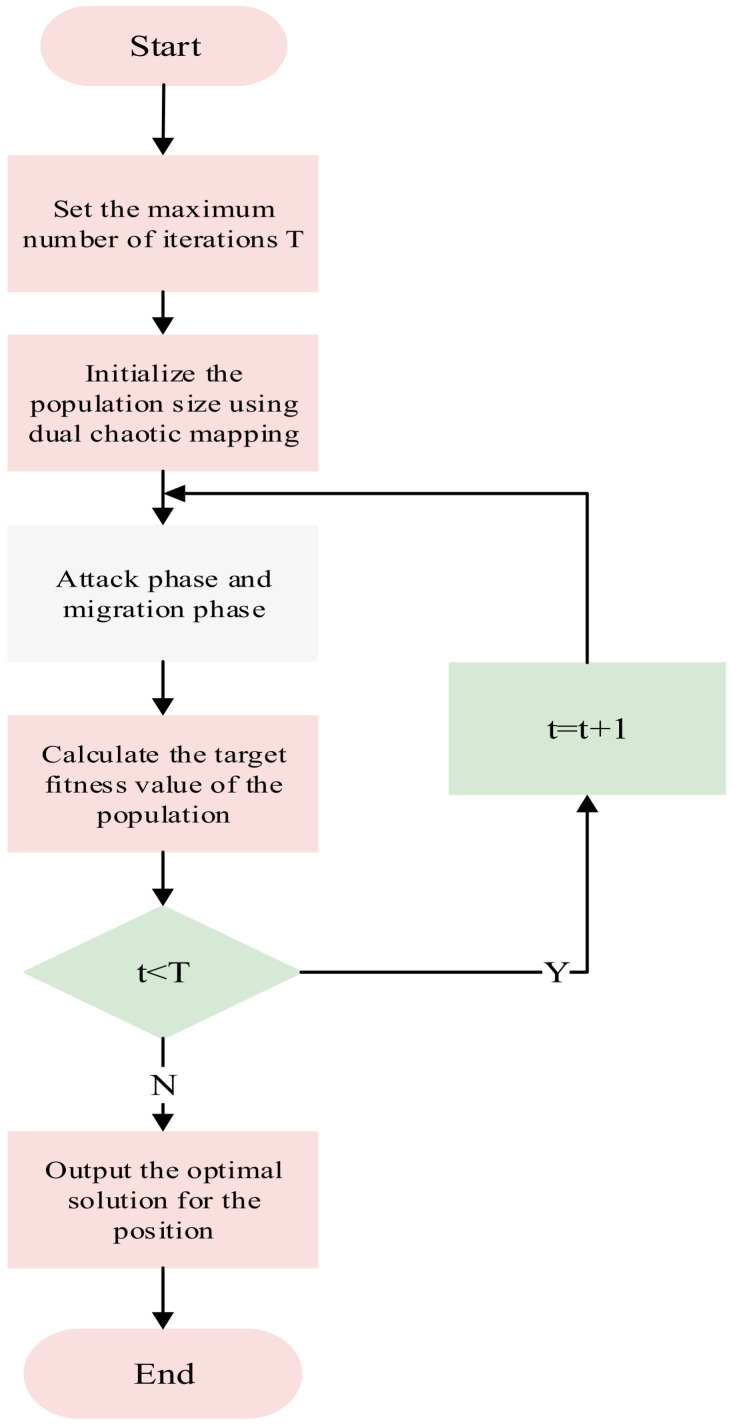
Flowchart of IBKA.

The algorithm flow is shown in Figure 6. The implementation steps are as follows:

Step 1: Define algorithm parameters and initialize the population. Define population size N, maximum iteration count T, etc. Use the Logistic-Tent chaotic mapping strategy to generate high-quality initial populations, ensuring reasonable population quantity and uniform spatial distribution to enhance population diversity and randomness.

Step 2: In the attack phase, different attack behaviors are used for global search. As the iteration count increases, update position states according to formulas to avoid local search entrapment and expand the search range.

Step 3: In the migration phase, update individual positions using the Levy flight strategy. Compare the fitness values of the current population and a random population. In each iteration, perform Levy flight operations on a portion of individuals with a certain probability to escape local regions. Update individual positions according to Equations (18) and calculate the current population’s fitness values.

Step 4: Determine whether termination conditions are met. During iterations, retain superior individuals and eliminate inferior ones. If termination conditions are satisfied, the algorithm terminates and outputs the current optimal fitness value and corresponding optimal solution. Otherwise, return to Step 2 for continued iterative optimization until termination conditions are met.

### 2.4. Effectiveness Analysis of IBKA

To validate the performance of the IBKA, 8 standard benchmark functions were selected for analysis. Additionally, 5 representative optimization algorithms were chosen for comparative evaluation, including:

Crested Porcupine Optimizer [26], Grey Wolf Optimizer [27], Whale Optimization Algorithm [28], Particle Swarm Optimization [29], Genetic Algorithm [30]. To ensure a thorough comparison among optimization algorithms, each algorithm was independently executed 50 times. All algorithms adopted the same population size of 10 and a maximum of 200 iterations. Eight standard benchmark functions, as shown in Table 1, including their mathematical formulations and value ranges, were employed to evaluate performance, with all functions sharing identical upper and lower bounds for dimensions. Functions F1–F4 are unimodal, while F5–F8 are multimodal, enabling comprehensive assessment of algorithmic behavior from multiple aspects. The optimization results of seven algorithms on these eight test functions are summarized in Table 2.

**Figure 7:**
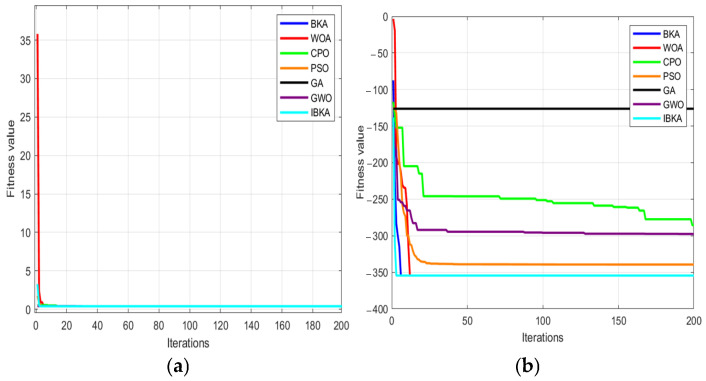
IBKA Optimization Results. (**a**) Optimization results of the algorithm on unimodal functions. (**b**) Optimization results of the algorithm on multimodal functions.

The F1–F4 functions are unimodal with only one global optimum. As observed in Table 2, the IBKA demonstrates significantly superior performance across all four unimodal test functions compared to the other six competing algorithms, achieving the highest convergence precision. The F5–F8 functions are multimodal, containing multiple local optima. The experimental results show that IBKA outperforms its counterparts in terms of global search capability. Two representative functions—the typical unimodal F2 and the complex multimodal F5—were selected for detailed analysis. As shown in Figure 7 (where subplot (a) corresponds to F2 and subplot (b) to F5), IBKA exhibits markedly faster convergence speed and higher search efficiency than the comparative algorithms.

## 3. FMD-IBKA-BTGN Model

**Figure 8:**
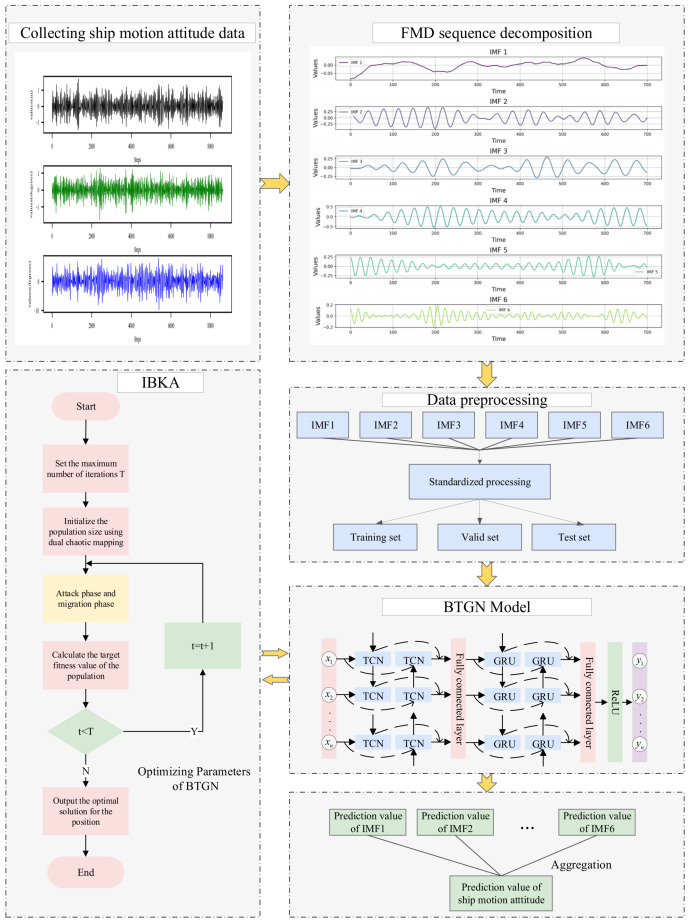
Model prediction process.

Due to the complex marine environment, the ship’s motion attitude exhibits dynamic time-varying and nonlinear characteristics, making it impossible for a single model to accurately predict ship motion attitudes. The proposed model conducts in-depth research on the impulsiveness and periodicity of ship motion attitudes, effectively handling nonlinear data and improving prediction accuracy. The prediction process of ship motion attitudes is shown in Figure 8.

By applying FMD, the original ship motion attitude data is decomposed into multiple intrinsic mode functions. These intrinsic mode functions are then divided into independent training sets, validation sets, and test sets. Subsequently, for each intrinsic mode function in the training set, the model is trained, and during the training process, the optimal hyperparameters of the model are searched and determined. Then, using these trained models, predictions are made for the corresponding intrinsic mode functions in the test set. Finally, all prediction results are reconstructed to obtain the final prediction results.

## 4. Experimental Analysis

### 4.1. Experimental Environment

The ship motion attitude prediction experiment was implemented using Python 3.9.13 within the PyTorch 2.0.0 framework, leveraging a GTX 3050 M GPU to accelerate deep learning computations. The development was conducted in the PyCharm 2022.3.2 integrated environment under Windows 11.

**Figure 9:**
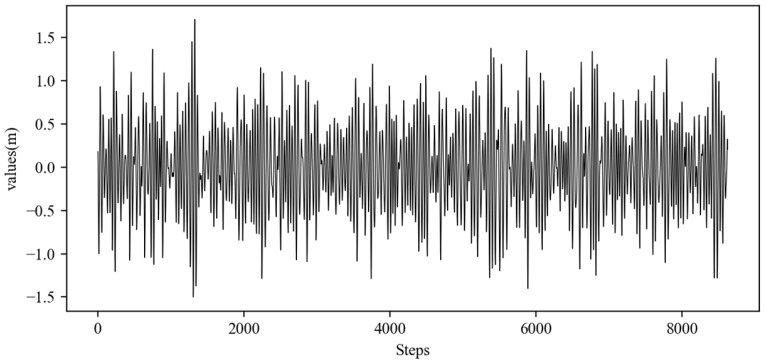
Raw data of ship heave motion.

The research conducted simulation experiments based on real-world measurement data. The data originate from observations of ship motion attitude for a real vessel navigating at 24 kn in sea state 4. Detailed ship parameters are listed in Table 3. The study collected continuous motion attitude data through an inertial measurement unit installed on the ship. The total duration was 1400 s, the sampling period was set to 0.1758 s, and 8000 sample data points were obtained. The data contained neither noise nor outliers, and the original ship heave motion data are shown in Figure 9.

### 4.2. Data Preprocessing

The dataset is divided into training set, validation set, and test set according to a 7:2:1 ratio. Prior to model training, a standardization process is applied to the data, as shown in the following formula:

(19) $$X_{std}=\frac{X-X_{mean}}{X_{\sigma }}$$

where X represents the actual data, $X_{mean}$ denotes the mean of the actual data, and $X_{\sigma }$ signifies the standard deviation of the actual data.

### 4.3. Evaluation Metrics

Mean Absolute Error (MAE), Mean Squared Error (MSE), Root Mean Squared Error (RMSE), and Mean Absolute Percentage Error (MAPE) are commonly used evaluation metrics in prediction tasks, where smaller values indicate better model predictive capability. *R*^2^ represents the fitting degree between actual and predicted values, and the closer the value is to 1, the stronger the model’s predictive capability. Using a single evaluation metric often has limitations, and comprehensively measuring multiple evaluation metrics can provide a deeper analysis of model performance. The formulas for the aforementioned five evaluation metrics are as follows:

(20) $$\mathrm{MAE}=\frac{1}{n}\sum_{i=1}^{n}\left|y_{i}-{\tilde{y}}_{i}\right|$$

(21) $$\mathrm{MSE}=\frac{1}{n}\sum_{i=1}^{n}{\left|y_{i}-{\tilde{y}}_{i}\right|}^{2}$$

(22) $$\mathrm{RMSE}=\sqrt{\frac{1}{n}\sum_{i=1}^{n}{\left|y_{i}-{\tilde{y}}_{i}\right|}^{2}}$$

(23) $$\mathrm{MAPE}=\frac{1}{n}\sum_{i=1}^{n}\frac{\left|y_{i}-{\tilde{y}}_{i}\right|}{y_{i}}\times 100\%$$

(24) $$R^{2}=1-\frac{\sum_{i=1}^{n}{\left(y_{i}-{\tilde{y}}_{i}\right)}^{2}}{\sum_{i=1}^{n}{\left(y_{i}-\bar{y}\right)}^{2}}$$

where $y_{i}$ represents the actual value at time t, ${\tilde{y}}_{i}$ denotes the predicted value at time *t*, $\bar{y}$ indicates the average value of the data, and n signifies the number of actual values.

### 4.4. IBKA Optimization of Model Parameters

**Figure 10:**
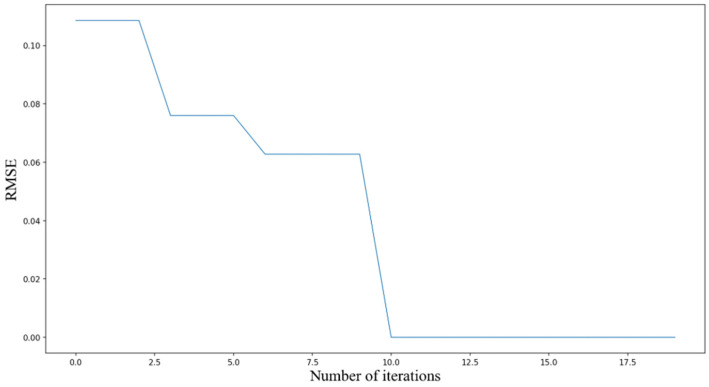
Optimization result descent process.

The IBKA primarily optimizes the following parameters in the model: training iterations, batch_size, TCN filter count, filter kernel size, and GRU hidden layer units, aiming to identify the optimal parameter combination. IBKA is applied to optimize the model parameters with an initial population size of 15, maximum iteration count of 20, and 2 optimized parameters. The parameter selection ranges are detailed in Table 4. The RMSE of the validation set serves as the optimization objective, targeting the minimization of validation RMSE. The RMSE reduction results during the IBKA parameter optimization process are shown in Figure 10.

From Figure 10, it can be observed that as the optimization algorithm IBKA iterates, the RMSE shows a gradual decreasing trend, which actually reflects IBKA’s ability to progressively improve model accuracy through the optimization process. In the first six iteration steps of the algorithm, due to the random assignment of model parameters, the decrease in RMSE is particularly significant, a phenomenon that further emphasizes the importance of high-quality parameters for enhancing the precision of neural network models. After 10 iterations, the RMSE has become very close to 0, indicating that the deviation between the experimental model’s prediction results and the true values has disappeared. IBKA demonstrates outstanding performance in optimizing model parameters, significantly improving model precision.

After the IBKA optimization process, the parameter settings for BTGN are shown in Table 5.

### 4.5. Experimental Result Analysis

#### 4.5.1. Selection and Comparison of Predictive Steps

To achieve short-term data updates, a sliding window of size w with stride s is defined. For historical ship motion attitude data $\left\{x_{1},x_{2},x_{3},\ldots ,x_{t}\right\}$, continuous prediction is implemented through this sliding window approach. Starting from time step t, subsequence $\left\{x_{t},x_{t+1},\ldots ,x_{t+w-1}\right\}$ is generated. The sliding window moves according to the stride length until the entire sequence update is completed.

#### 4.5.2. FMD

**Figure 11:**
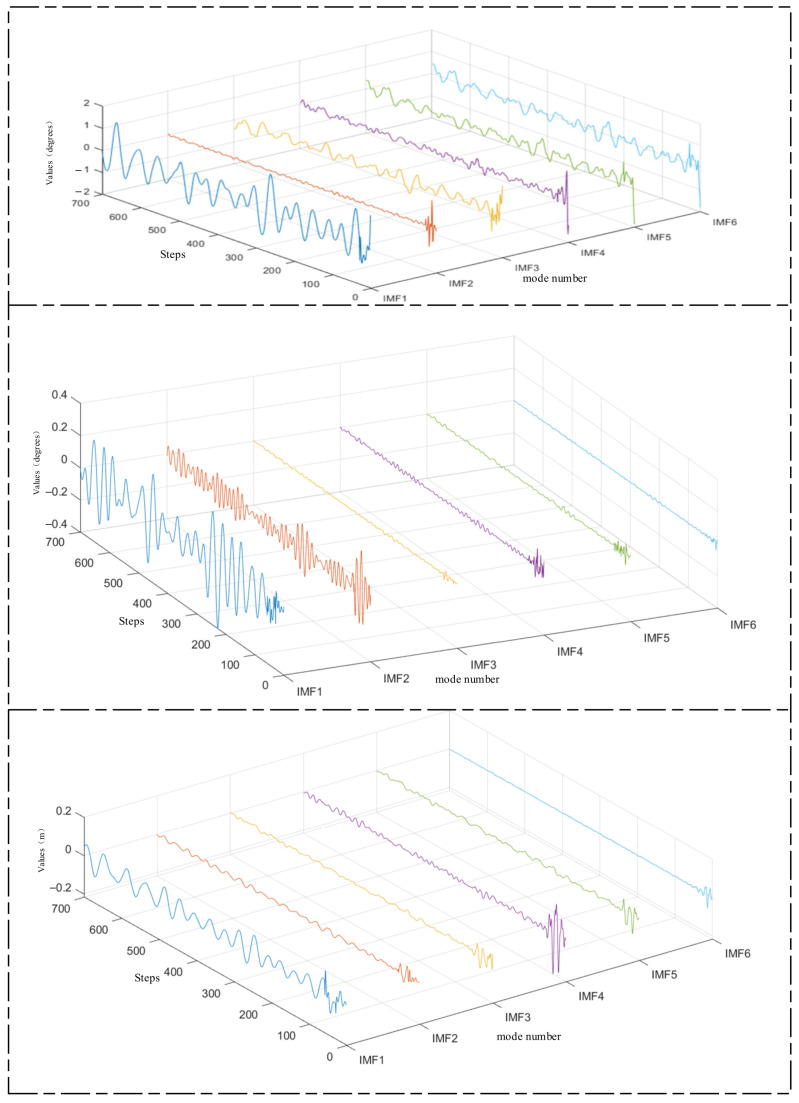
Decomposition of roll, pitch, and heave.

Firstly, FMD is applied to decompose the roll, pitch, and heave data. Subsequently, the BTGN model is used to train and predict each decomposed component. Finally, the predicted results are reconstructed and integrated to obtain the final outcome. Figure 11 illustrates the decomposition of roll, pitch, and heave data using FMD.

**Figure 12:**
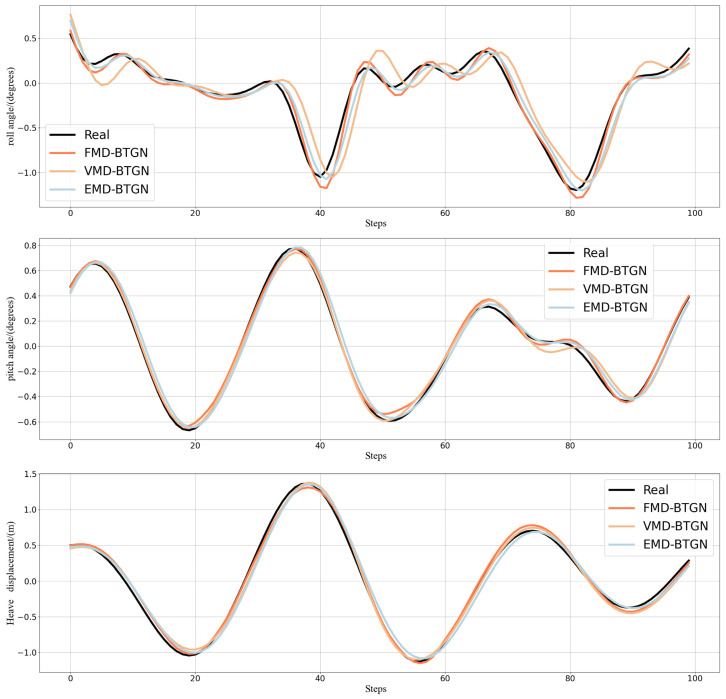
Prediction of roll, pitch, and heave in Sea State 4.

FMD fully considers the periodic and impulsive characteristics of ship motion attitude data. It maintains sensitivity to prominent features while demonstrating robustness against interference and noise. FMD employs an adaptive filter decomposition mode that is unrestricted by filter shape, bandwidth, or center frequency. After FMD, the fluctuation amplitudes of high-frequency and mid-frequency components are relatively small, with most values falling within the range of [−0.2, 0.2]. This indicates a more thorough decomposition of high-frequency and mid-frequency components. In this section, we compare FMD-BTGN, VMD-BTGN, and EMD-BTGN. The primary parameters for FMD, VMD, and EMD are provided in Table 6. The comparison results are shown in Table 7 and Figure 12.

Comparing the prediction results of the three methods, the FMD-BTGN model demonstrates closer alignment with the actual data compared to VMD-BTGN and EMD-BTGN. Additionally, the values of MAE, MSE, RMSE, and MAPE are significantly reduced, and *R*^2^ is closer to 1, validating the effectiveness of FMD in data decomposition and enhancing the prediction accuracy of the base model.

#### 4.5.3. Experimental Comparison

This paper compares the FMD-IBKA-BTGN model with the BTGN and FMD-BKA-BTGN models to verify the effectiveness of the hybrid model. Subsequently, the FMD-IBKA-BTGN model is compared with other existing methods to validate its predictive performance. The model parameter settings are shown in Table 8. The input sequence length for all models is set to 10, while the prediction step length remains constant. Each model uses the same activation function and optimizer. The first 700 data points in the dataset are allocated for training across all models, while the remaining data are used for testing. A total of 50 experiments are conducted under identical experimental conditions and model parameter settings, and the average of the 50 sets of prediction error data is taken. During the testing phase, the model parameters remain fixed and rely solely on information learned from the training set. The prediction errors of the FMD-IBKA-BTGN model compared with other existing methods are presented in Table 9.

In Table 9, the FMD-IBKA-BTGN model demonstrates significant performance improvement compared to other models. Specifically, this model achieves notable reductions in four key evaluation metrics: MSE, MAE, RMSE, and MAPE. Particularly when compared to the LSTM baseline model, the FMD-IBKA-BTGN model reduces MSE by an average of 0.97%, MAE by 4.2%, RMSE by 7.4%, and MAPE by 20.38%. The FMD-IBKA-BTGN model exhibits superior predictive accuracy, with enhanced fitting precision and generalization capability. Therefore, the improved model can more accurately capture and reflect changes in ship motion attitude.

**Figure 13:**
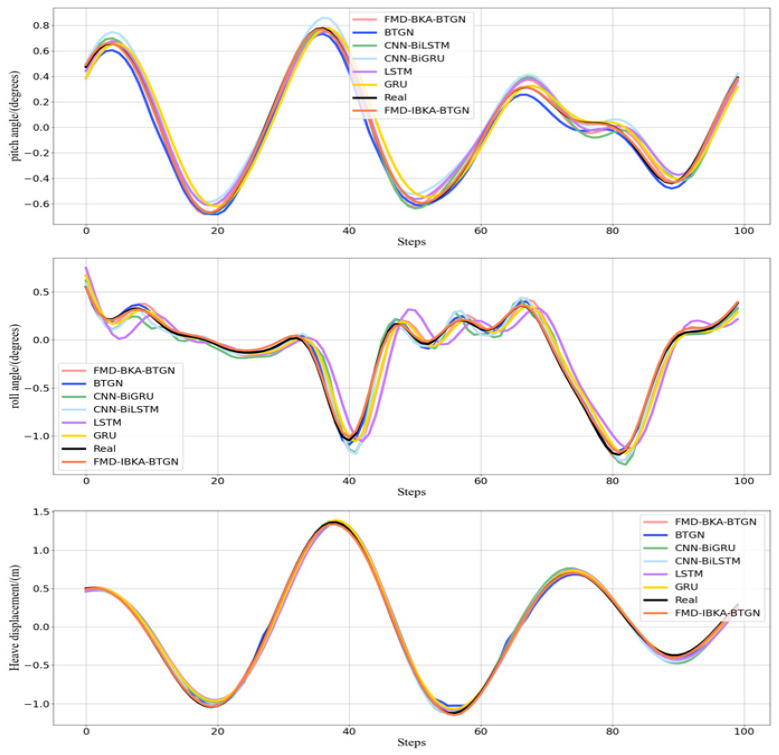
Different model prediction of roll, pitch, and heave in Sea State 4.

The curve comparison plots of prediction data and actual data from the seven models are shown in Figure 13. Further analysis reveals that the state sequence of ship motion attitudes exhibits imbalanced distribution characteristics and dynamic temporal variation. After decomposing ship motion data using FMD, not only is the complex data effectively decomposed, but subsequent models processing the decomposed data can capture more refined and critical information. When optimizing BTGN parameters using IBKA with RMSE as the fitness function, the model’s prediction capability is significantly enhanced.

The prediction results of the BTGN, FMD-BTGN, and FMD-BKA-BTGN models are all inferior to those of the FMD-IBKA-BTGN model. The prediction result distribution of the FMD-IBKA-BTGN model is closer to the original sequence distribution compared to BTGN, FMD-BTGN, and FMD-BKA-BTGN. Compared to BTGN, both CNN-BiLSTM and CNN-BiGRU possess bidirectional features, but CNN has limited capability in extracting temporal sequence features. The BTGN model’s structure better considers forward and backward data characteristics, demonstrating stronger feature extraction capability. Although these models can reflect the trend of ship motion attitude changes, when facing non-stationary data and continuous sample updates, the FMD-IBKA-BTGN model exhibits stronger adaptability. Other models only achieve high prediction accuracy under relatively stable data conditions. The FMD-IBKA-BTGN model, by thoroughly decomposing data and adaptively adjusting model parameters based on data characteristics, maintains high adaptability between model architecture and data throughout the prediction cycle. Therefore, the FMD-IBKA-BTGN model demonstrates higher prediction accuracy and robustness in handling complex time-series tasks.

**Figure 14:**
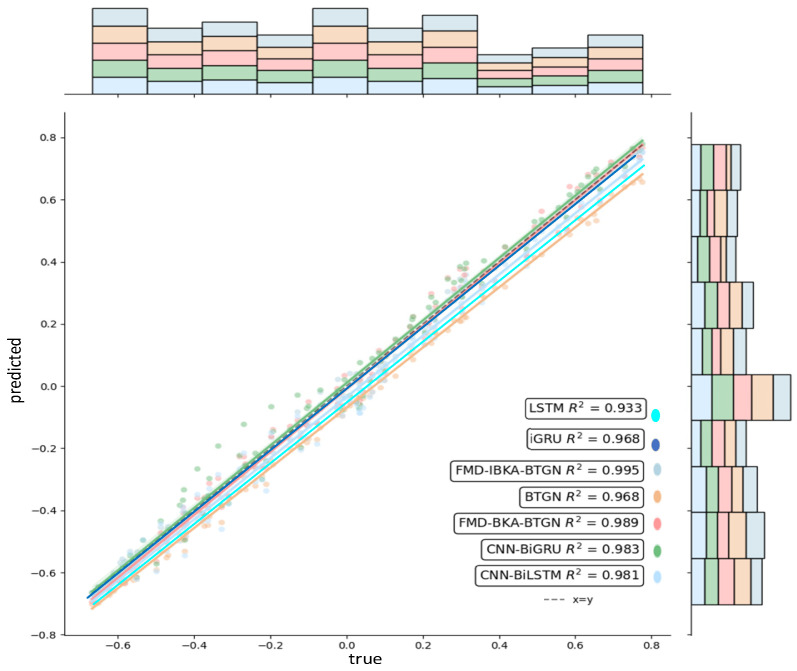
Comparison of pitch fitting.

**Figure 15:**
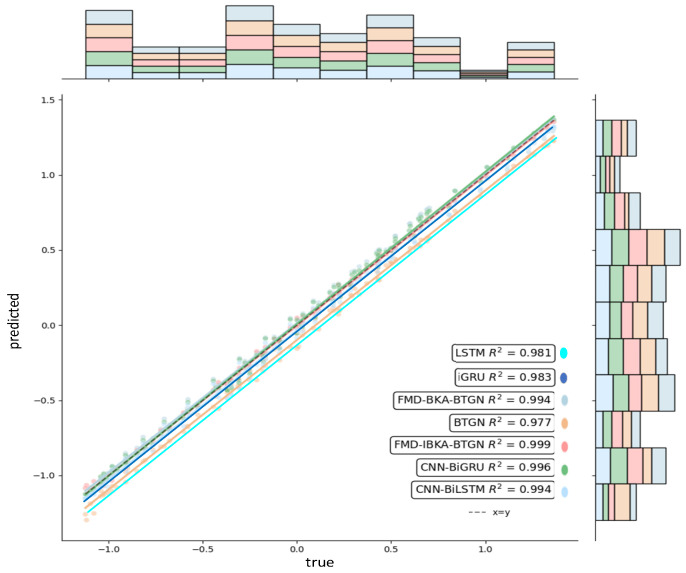
Comparison of roll fitting.

**Figure 16:**
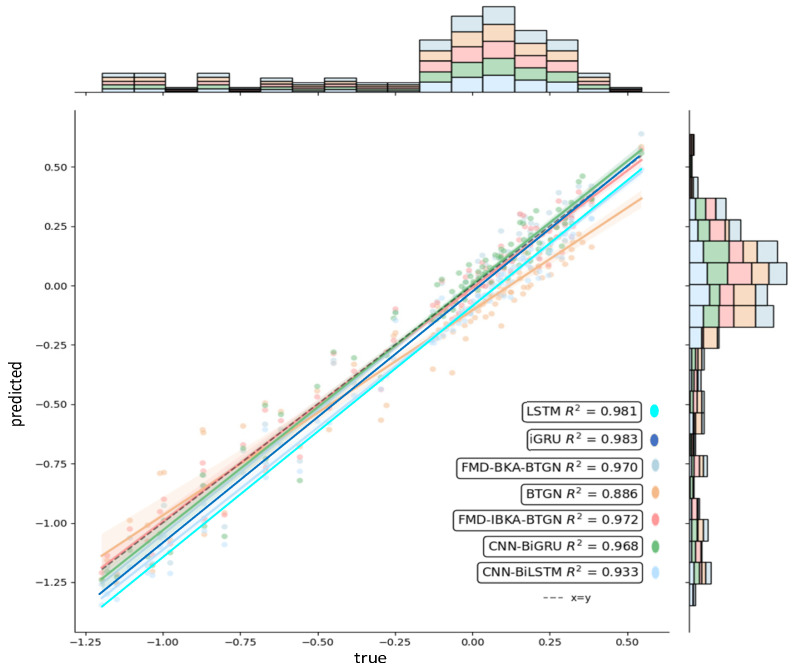
Comparison of heave fitting.

**Figure 17:**
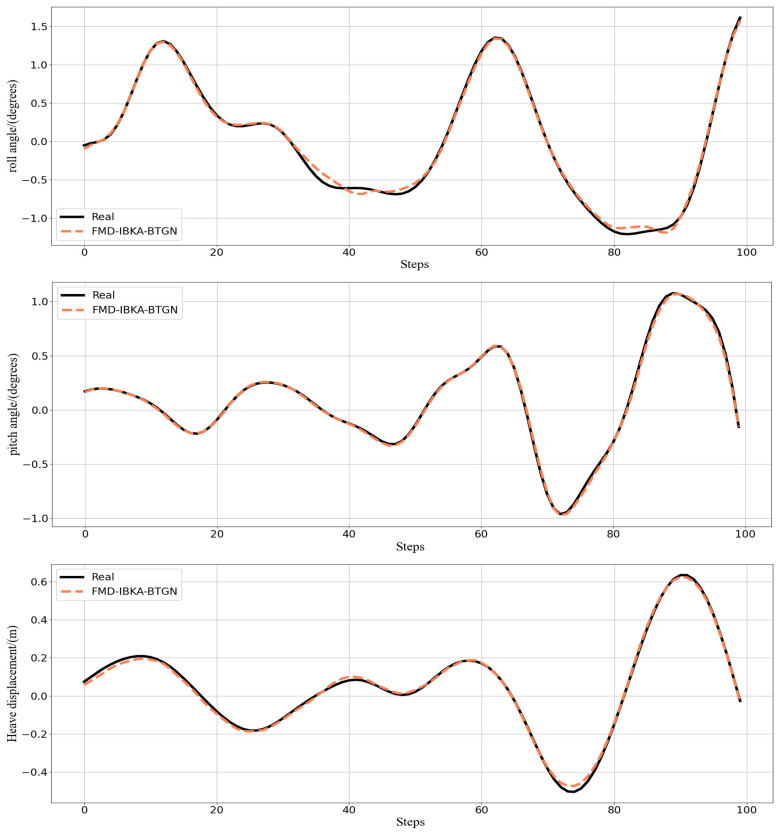
FMD-IBKA-BTGN model prediction results in Sea State 5.

**Figure 18:**
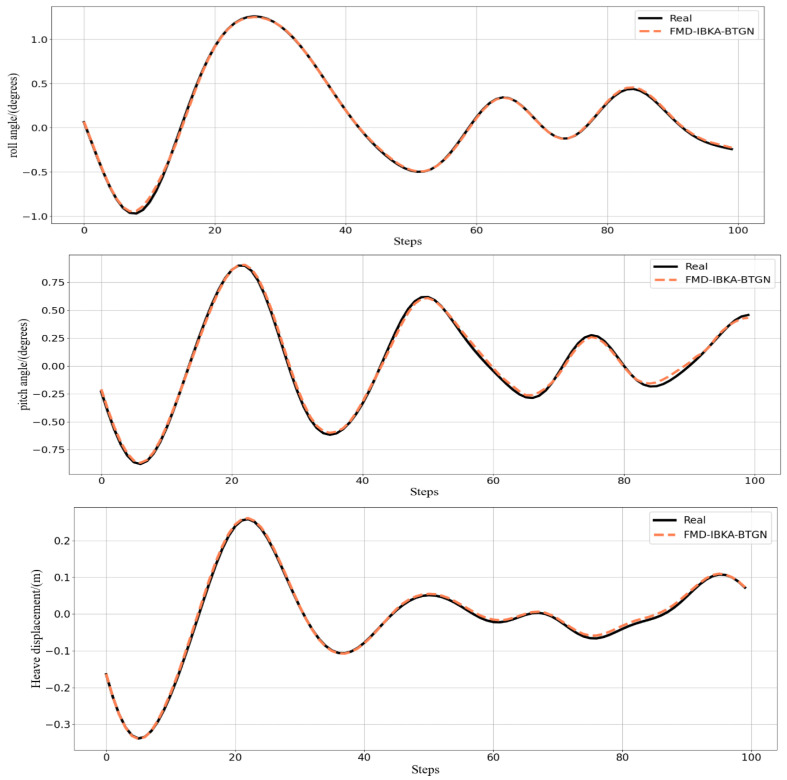
FMD-IBKA-BTGN model prediction results in Sea State 6.

The comparison results of the model’s coefficient of determination *R*^2^ are shown in Figure 14, Figure 15 and Figure 16, further demonstrating the fitting capability between predicted and actual values. As observed in the figures, the proposed model exhibits *R*^2^ values closer to 1 compared to other models, proving its superiority in prediction accuracy with good fitting precision and generalization capability. However, as sea conditions deteriorate, wave effects on ship motion attitudes become significant, rendering the data highly non-stationary and challenging to predict. To validate performance under extreme conditions, operational data from a vessel navigating in Sea State 5 (90° encounter angle at 20 knots) and Sea State 6 (120° encounter angle at 20 knots) were selected for predictive analysis. The prediction results of the FMD-IBKA-BTGN model are displayed in Figure 17 and Figure 18, showing a high consistency between predicted and actual motion data. This confirms the model’s universal applicability in ship motion attitude prediction, achieving accurate results across varying sea conditions.

In summary, the FMD-IBKA-BTGN model demonstrates significantly higher prediction accuracy across varying sea conditions and encounter angles compared to other models. The model’s advantages become more pronounced with increased prediction difficulty, maintaining consistent precision across different sea states while achieving extremely high performance throughout the prediction cycle. It effectively handles local peaks and abrupt data changes. By accurately forecasting ship motion attitude data, this model aids crew members in making informed decisions to ensure safe navigation.

## 5. Conclusions

This study proposes a novel hybrid intelligent model, FMD-IBKA-BTGN, for ship motion attitude prediction, which significantly enhances prediction accuracy and generalization capability under non-stationary sea conditions through a multi-stage collaborative optimization strategy. Unlike conventional approaches relying solely on single neural networks or empirical mode decomposition techniques, this work introduces systematic innovations at three levels—data preprocessing, hyperparameter optimization, and model architecture design—and validates its superior performance using real ship motion data.

In the signal decomposition phase, the proposed FMD method overcomes the inherent modal aliasing issues prevalent in traditional EMD-based approaches when processing oceanic motion signals characterized by high randomness and impulsive disturbances. By jointly extracting periodic and transient impulse components, FMD enables fine-grained decoupling of critical motion modes such as roll, pitch, and heave. Experimental results demonstrate that IMFs derived from FMD exhibit enhanced physical interpretability, providing clearer time-scale structural information essential for downstream modeling tasks.

To address the high parameter sensitivity typical of deep models, this study designs an IBKA, incorporating a Logistic-Tent dual chaotic mapping for population initialization and integrating Lévy flight mechanisms to enhance global search capabilities. Compared with classical optimizers such as PSO, GWO, and standard BKA, IBKA exhibits faster convergence and a stronger ability to escape local optima.

The proposed BTGN combines the advantages of Bi TCN in capturing local temporal patterns with the advantages of Bi GRU in modeling long-term dependencies. Their combination enables the model to simultaneously perceive short-term transients and long-term trends in ship motion. The IBKA further optimized the parameters of the BTGN model, thereby improving the prediction accuracy of the hybrid model.

From an engineering perspective, the proposed model exhibits strong potential for onboard deployment. It can be integrated into shipborne navigation systems to enable real-time short-term forecasts, supporting autonomous decision-making, anti-roll control, cargo safety monitoring, and route planning for adverse weather avoidance. Nonetheless, limitations remain, input variables are restricted to motion signals alone, excluding explicit environmental factors such as wind speed, wave height, and current fields, which constrains cross-sea-state transferability.

Future work will incorporate real-time onboard sensors—including meteorological stations, radar wave spectrometers, and AIS—to build a multi-source heterogeneous data-driven framework, exploring environment-motion coupled modeling mechanisms. Additionally, efforts will focus on network lightweighting and edge computing deployment strategies to minimize computational latency, advancing the model toward practical closed-loop control systems.

## Figures and Tables

**Table 1:** Objective function formula and value range.

Number	Formula	Value Interval	Theoretical Optimum
F1	$f\left(x\right)=\max\left(\left|x\right|\right)$	$\left[-100,100\right]$	0
F2	$f(x)=\sum \left(100\cdot {\left(x_{2}-x_{1}^{2}\right)}^{2}+{\left(x_{1}-1\right)}^{2}\right)$	$\left[-30,30\right]$	0
F3	$f\left(x\right)={\sum \left(\left\lfloor x+0.5\right\rfloor \right)}^{2}$	$\left[-100,100\right]$	0
F4	$f\left(x\right)=\sum x_{i}^{2}$	$\left[-100,100\right]$	0
F5	$f(x)=\sum \left(-x\cdot \sin\left(\sqrt{\left|x\right|}\right)\right)$	$\left[-50,50\right]$	−12,569.5
F6	$f(x)=\sum \left[x^{2}-10\cos(2\pi x)+10\right]$	$\left[-10,10\right]$	0
F7	$f\left(x\right)=\frac{1}{4000}\sum x^{2}-\prod \cos\left(\frac{x}{\sqrt{i}}\right)+1$	$\left[-600,600\right]$	0
F8	$f(x)=-20\exp\left(-0.2\sqrt{\frac{1}{n}\sum_{i=1}^{n}x_{i}^{2}}\right)-\exp\left(\frac{1}{n}\sum_{i=1}^{n}\cos(2\pi x_{i})\right)+20+e$	$\left[-32,32\right]$	0

**Table 2:** Optimization Results of Benchmark Functions.

Number	IBKA	BKA	WOA	CPO	PSO	GA	GWO
F1	4.84 × 10^−20^	7.65 × 10^−17^	52.1821	5.70976	1.37535	61.9362	5.51 × 10^−6^
F2	0.0001	0.00486	0.54084	0.00679	3.58147	3.15346	0.60223
F3	1.73 × 10^−7^	0.00017	0.40455	0.00325	2.54143	205.192	0.72262
F4	0.0	0.0	0.00608	0.00256	2.56298	2161.99	2.49 × 10^−7^
F5	−350.92	−329.59	−340.24	−259.69	−310.98	−125.691	−279.011
F6	0.0	0.0	2.39 × 10^−14^	49.33893	156.8184	227.1569	4.16582
F7	0.0	0.0	0.00431	0.08721	0.267312	120.2302	0.003422
F8	3.44 × 10^−16^	6.99 × 10^−16^	3.45 × 10^−14^	2.67231	2.975234	19.75423	1.25 × 10^−12^

**Table 3:** Main Parameters of Ships.

Parameter	Value
Length Overall (m)	240
Beam (m)	45
Draft (m)	8
Displacement (t)	32,000

**Table 4:** Range of Parameter Values.

Parameter	Value Interval
epoch	[20, 100]
batch_size	[8, 128]
nb_filters	[16, 128]
kernel_size	[2, 10]
hidden_size	[10, 200]

**Table 5:** Parameter values after optimization.

Parameter	Value Interval
epoch	42
batch_size	12
nb_filters	87
kernel_size	5
hidden_size	50

**Table 6:** Parameter values for different decomposition methods.

Method	Items	Values
FMD	filtersize	30
	cutnum	7
	mode number	6
VMD	penalty Coefficient α	2000
	mode number k	6
EMD	termination conditions $threshold_{1}$	0.01
	termination conditions $threshold_{2}$	0.1
	tolerance	0.01

**Table 7:** Comparison of prediction errors.

Method		FMD-BTGN	VMD-BTGN	EMD-BTGN
MSE	roll	0.0053	0.0363	0.0096
	pitch	0.0009	0.0019	0.0026
	heave	0.0027	0.0039	0.0061
MAE	roll	0.0569	0.1492	0.0752
	pitch	0.0247	0.0346	0.0440
	heave	0.0456	0.0519	0.0675
RMSE	roll	0.0728	0.1906	0.0984
	pitch	0.0296	0.0436	0.0512
	heave	0.0520	0.0630	0.0784
MAPE	roll	68.5696	140.871	71.9932
	pitch	29.8029	35.3957	43.3955
	heave	35.0247	56.3129	69.6440
$R^{2}$	roll	0.9071	0.7965	0.8742
	pitch	0.9442	0.9223	0.9173
	heave	0.8978	0.8964	0.8876

**Table 8:** Different model parameter values.

Method	Items	Values
BTGN	epoch	50
	batch_size	32
	nb_filters	64
	kernel_size	4
	hidden_size	128
FMD-IBKA-BTGN	epoch	42
	batch_size	12
	nb_filters	87
	kernel_size	5
	hidden_size	50
CNN-BiGRU/CNN-BiLSTM/GRU/LSTM	epoch	50
	Batch_size	32
	filters	64
	kernel_size	2
	hidden_size	128

**Table 9:** Comparison of evaluation indicators for prediction models.

		FMD-IBKA-BTGN	FMD-BKA-BTGN	BTGN	CNN-BiLSTM	CNN-BiGRU	GRU	LSTM
MSE	roll	0.0001	0.0050	0.0203	0.0119	0.0057	0.0125	0.0132
	pitch	0.0001	0.0018	0.0053	0.0032	0.0028	0.0047	0.0057
	heave	0.0002	0.0004	0.0106	0.0028	0.0016	0.0031	0.0108
MAE	roll	0.0065	0.0548	0.1277	0.0949	0.0056	0.0964	0.0654
	pitch	0.0035	0.0037	0.0696	0.0456	0.0407	0.0674	0.0503
	heave	0.0231	0.0276	0.0981	0.0432	0.0341	0.0541	0.0438
RMSE	roll	0.0140	0.0710	0.1425	0.1089	0.0755	0.1118	0.1149
	pitch	0.0140	0.0431	0.0733	0.0569	0.0527	0.0686	0.0755
	heave	0.0447	0.0220	0.1032	0.0532	0.0406	0.0557	0.1039
MAPE	roll	10.0756	19.1781	48.6832	30.7462	28.9219	33.6735	35.4219
	pitch	11.3568	15.4987	20.8804	25.5054	22.0030	19.4521	21.9648
	heave	12.6535	16.5483	36.2201	22.9875	20.4851	25.7438	37.8527

## Data Availability

The raw data supporting the conclusions of this article will be made available by the authors on request.

## References

[1] Su Z.Q., Wu C.L., Xiao Y.J., He H.D. (2022). Study on the prediction model of accidents and incidents of cruise ship operation based on machine learning. Ocean Eng..

[2] Garcia-Huerta R.A., González-Jiménez L.E., Villalon-Turrubiates I.E. (2020). Sensor fusion algorithm using a model-based Kalman filter for the position and attitude estimation of precision aerial delivery systems. Sensors.

[3] Tang G., Yao X.Q., Li F.R., Wang Y.D., Hu X. (2022). Prediction about the vessel’s heave motion under different sea states based on hybrid PSO-ARMA model. Ocean Eng..

[4] Chen M.Z., Challita U., Saad W., Yin C.C., Debbah M. (2019). Artificial neural networks-based machine learning for wireless networks: A tutorial. IEEE Commun. Surv. Tut..

[5] Suhermi N., Prastyo D.D., Ali B. (2018). Roll motion prediction using a hybrid deep learning and ARIMA model. Procedia Comput. Sci..

[6] Suo Y., Chen W., Claramunt C., Yang S. (2020). A ship trajectory prediction framework based on a recurrent neural network. Sensors.

[7] Wang X., Liu J., Peng H., Qie X., Zhao X., Lu C. (2022). A simultaneous planning and control method integrating APF and MPC to solve autonomous navigation for USVs in unknown environments. J. Intell. Robot. Syst..

[8] Tang G., Lei J.M., Shao C.T., Hu X., Cao W.D., Men S. (2021). Short-term prediction in vessel heave motion based on improved LSTM model. IEEE Access.

[9] Sun P., Boukerche A., Tao Y.J. (2020). SSGRU: A novel hybrid stacked GRU-based traffic volume prediction approach in a road network. Comput. Commun..

[10] Zhang D., Chu X., Liu C., He Z., Zhang P., Wu W. (2024). A review on motion prediction for intelligent ship navigation. J. Mar. Sci. Eng..

[11] Wang H.F., Yin J.C., Wang N.N., Wang L.J. (2025). A multi-dimensional data-driven ship roll prediction model based on VMD-PCA and IDBO-TCN-BiGRU-Attention. Front. Mar. Sci..

[12] Zhang L., Feng X.C., Wang L., Gong B.M., Ai J.L. (2024). A hybrid ship-motion prediction model based on CNN-MRNN and IADPSO. Ocean Eng..

[13] Su Y., Lin J., Zhao D., Guo C., Wang C., Guo H. (2020). Real-time prediction of large-scale ship model vertical acceleration based on recurrent neural network. J. Mar. Sci. Eng..

[14] Ullah S., Kim D.H. (2020). Lightweight driver behavior identification model with sparse learning on in-vehicle CAN-bus sensor data. Sensors.

[15] Zhao J.X., Zhao Y. (2024). An enhanced model based on deep operator network for very short-term forecasting of ship motion. Phys. Fluids.

[16] Peng X.Y., Zhang B. (2019). Ship motion attitude prediction based on EMD-PSO-LSTM combined model. J. Chin. Inert. Technol..

[17] Chen Y., Sun B., Xie X., Li X., Li Y., Zhao Y. (2024). Short-term forecasting for ship fuel consumption based on deep learning. Ocean Eng..

[18] Zhou T., Yang X., Ren H., Li C., Han J. (2023). The prediction of ship motion attitude in seaway based on BSO-VMD-GRU combination model. Ocean Eng..

[19] Miao Y., Zhang B., Li C., Lin J., Zhang D. (2022). Feature mode decomposition: New decomposition theory for rotating machinery fault diagnosis. IEEE Trans. Ind. Electron..

[20] Zhang B., Wang S., Deng L., Jia M., Xu J. (2023). Ship motion attitude prediction model based on IWOA-TCN-Attention. Ocean Eng..

[21] Li Y., Shi B., Qiao W., Du Z. (2025). A black-winged kite optimization algorithm enhanced by osprey optimization and vertical and horizontal crossover improvement. Sci. Rep..

[22] Li C., Zhang K., Zheng B., Chen Y. (2025). Path planning problem solved by an improved black-winged kite optimization algorithm based on multi-strategy fusion. Int. J. Mach. Learn. Cybern..

[23] Zhang Z., Wang X., Yue Y. (2024). Heuristic optimization algorithm of Black-Winged Kite fused with osprey and its engineering application. Biomimetics.

[24] Li M.W., Xu R.Z., Geng J., Hong W.C., Li H. (2024). A ship motion forecasting approach based on Fourier transform, regularized Bi-LSTM and chaotic quantum adaptive WOA. Ocean Eng..

[25] Gao N., Chuang Z., Hu A. (2024). Real-Time Prediction of Ship Motion Based on Improved Empirical Mode Composition and Dynamic Residual Neural Network. Ocean Eng..

[26] Abdel-Basset M., Mohamed R., Abouhawwash M. (2024). Crested Porcupine Optimizer: A New Nature-Inspired Metaheuristic. Knowl.-Based Syst..

[27] Mirjalili S., Lewis A. (2014). Grey Wolf Optimizer. Adv. Eng. Softw..

[28] Mirjalili S., Lewis A. (2016). The Whale Optimization Algorithm. Adv. Eng. Softw..

[29] Shami T.M., El-Saleh A.A., Alswaitti M., Al-Tashi Q., Summakieh M.A., Mirjalili S. (2022). Particle swarm optimization: A comprehensive survey. IEEE Access.

[30] Katoch S., Chauhan S., Kumar V. (2021). A Review on Genetic Algorithm: Past, Present, and Future. Multimed. Tools Appl..

